# Multiscale Design and Simulation of CdSe/ZnS/MoTe_2_ Hybrid Photodetectors

**DOI:** 10.3390/s26082516

**Published:** 2026-04-19

**Authors:** Saddam Hussain, Yuxin Liu, Nasrullah Wazir, Krishna Krishna, Li Tao

**Affiliations:** 1 State Key Laboratory of Chips and Systems for Advanced Light Field Display, and Center for Quantum Physics, Key Laboratory of Advanced Optoelectronic Quantum Architecture and Measurement (MOE), School of Physics, Beijing Institute of Technology, Beijing 100081, China; 2 School of Electronics and Communication Engineering, Quanzhou University of Information Engineering, Quanzhou 362000, China; 3 Department of Chemistry, Indian Institute of Technology Kharagpur, Kharagpur 721302, India

**Keywords:** MoTe_2_-based photodetector, CdSe/ZnS quantum dots, heterostructures, optoelectronic simulations

## Abstract

Two-dimensional MoTe_2_ is applicable for near-infrared photodetection; however, low absorption in the visible range limits its performance. One way to overcome these limitations is by hybridizing with light-absorbing nanomaterials. In this study, we simulate a CdSe/ZnS quantum dot (QD)-sensitized MoTe_2_ photodetector at the coupled electromagnetic and device level. COMSOL Multiphysics demonstrates that the heterostructure of MoTe_2_/CdSe/ZnS on a SiO_2_/Si substrate exhibits a broadband-visible enhancement in absorption due to QD exciton absorption and Fabry–Perot interferences in the silicon dioxide layer. A staggered type-I band alignment of the CdSe/ZnS/MoTe_2_ interface was confirmed by COMSOL analysis, which also permits interfacial charge separation. Simulations of QD integration by Silvaco technology computer-aided design reveal that QD integration increases photocurrent through photogating and carrier transfer. The optimized device has a responsivity and detectivity of 1.3 × 10^−3^, 2 × 10^−3^ A/W, 9.4 × 10^8^, and 1.34 × 10^9^ Jones, and an external quantum efficiency of 0.31% and 0.394% at 520 and 630 nm, respectively, which is significantly better than pristine MoTe_2_ photodetectors. These results demonstrate the potential of CdSe/ZnS/MoTe_2_ heterostructures for high-performance broadband photodetection and establish a framework for correlating multiscale simulations with material properties and device performance.

## 1. Introduction

Photodetectors are critical components in advanced optoelectronic devices and are widely employed in optical communication, environmental monitoring, imaging, and biomedical diagnosis [1,2,3,4,5]. Traditional bulk semiconductors such as silicon (Si) and indium gallium arsenide (InGaAs) exhibit limited spectral tunability, high processing costs, and integration constraints, necessitating the exploration of new material platforms to improve key performance metrics, including responsivity, detectivity, and response time [6,7,8,9]. Two-dimensional (2D) transition metal dichalcogenides (TMDs), which exhibit strong light–matter interaction and tunable electronic properties, have been considered as potential materials for next-generation photodetectors [10,11,12,13,14,15,16]. Materials such as MoS_2_, WS_2_, WSe_2_, and MoSe_2_ have demonstrated broadband response and compatibility with flexible and transparent electronics [17,18,19,20,21,22]. In addition, reconfigurable and non-volatile neuromorphic photovoltaic detectors based on 2D semiconductors have highlighted the potential of hybrid optoelectronic devices for integrating sensing, memory, and computation within a single platform [23]. Moreover, broadband multidimensional photodetectors employing metasurface-assisted heterostructures have demonstrated the feasibility of simultaneously enhancing wavelength selectivity, polarization sensitivity, and broadband response [24]. Among them, molybdenum telluride (MoTe_2_) is particularly attractive due to its narrow bandgap, which enables efficient absorption extending into the near-infrared (NIR) region and supports broadband photodetection [25,26]. Furthermore, MoTe_2_ exhibits ambipolar transport and relatively high carrier mobility, making it suitable for field-effect transistor (FET)-based photodetectors [27,28,29]. Despite these advantages, pristine MoTe_2_ photodetectors often exhibit limited photoresponsivity in the visible region and degrade in performance due to trap-assisted recombination and insufficient optical absorption [30,31,32]. To overcome these limitations, hybrid heterostructure approaches have been widely explored, combining TMDs with complementary low-dimensional materials to enhance light absorption and carrier separation [33]. Zero-dimensional (0D) semiconductor quantum dots (QDs) are particularly attractive photosensitizers due to their large absorption cross-sections, size-tunable bandgap, and solution-processable fabrication [34,35]. Core–shell QDs such as CdSe/ZnS offer enhanced photostability and reduced surface trap densities, making them well suited for optoelectronic applications [36,37]. When integrated with 2D semiconductors, QDs can efficiently harvest incident photons and transfer photogenerated carriers across the interface, extending the spectral response and significantly enhancing photocurrent through photogating and charge transfer effects [38,39].

The literature reports that the hybrid graphene–QDs [40], MoS_2_-QDs [41], and WSe_2_-QDs [42] systems have demonstrated better performance in terms of responsivity and detectivity in the visible and NIR wavelengths. However, when it comes to MoTe_2_-based photodetectors, little is known in regard to how optical interference, excitonic absorption, band alignment, and carrier transport influence the operations of the devices. In particular, systematic studies of electromagnetic modeling, band structure analysis, and device-level transport simulations are rarely done. In this work, we report a detailed theoretical study of a CdSe/ZnS QDs-sensitized MoTe_2_ photodetector by combining optical simulations, density functional theory (DFT) calculations, and Silvaco technology computer-aided design (TCAD) device modeling. Band alignment analysis reveals a type-I heterostructure at the CdSe/ZnS/MoTe_2_ interface, which favors efficient charge separation. Device-level simulations prove that QD integration improves photocurrent and leads to strong photogating behavior and consequent large improvements in responsivity, detectivity, and external quantum efficiency. These results demonstrate CdSe/ZnS/MoTe_2_ heterostructures as a promising platform for high-performance broadband photodetection and give valuable design guidelines for hybrid low-dimensional optoelectronic devices.

## 2. Results and Discussion

### 2.1. Optical Properties of CdSe/ZnS QDs/MoTe_2_

**Figure 1:**
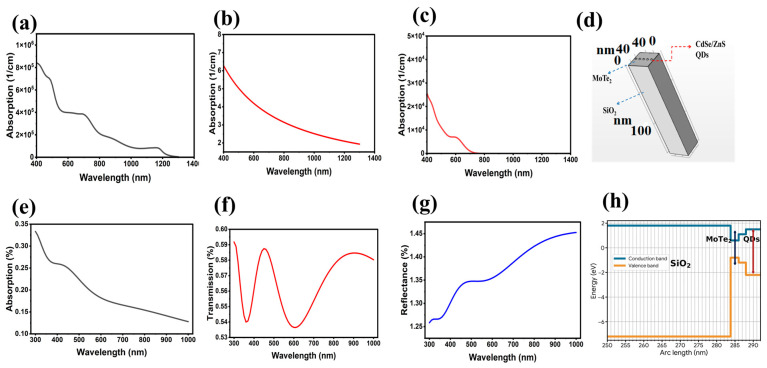
The COMSOL Multiphysics simulation output of the optical and electronic properties of the SiO_2_/MoTe_2_/CdSe/ZnS heterostructure. (**a**–**c**) The absorption spectrum of MoTe_2_, SiO_2_, and CdSe/ZnS QDs. (**d**) The multilayer geometry visualized using a COMSOL-based simulation. (**e**) Absorption, (**f**) transmission, and (**g**) reflection spectra of the complete SiO_2_/MoTe_2_/CdSe/ZnS. (**h**) Energy band diagram of the heterostructure that shows the alignment of type-I bands between the layers of MoTe_2_ and CdSe/ZnS and provides the possibility of carrier confinement and separation of charges. From left to right, the energy band diagram corresponds to SiO_2_ MoTe_2_/CdSe/ZnS. SiO_2_ acts as the insulating substrate, MoTe_2_ is the active layer, and CdSe/ZnS represents the core–shell quantum dots.

COMSOL Multiphysics (version 6.3) was used to simulate the optical properties using 3D geometry. SiO_2_ is used as a dielectric with a thickness of 285 nm. MoTe_2_ is used as an active medium with a size of 0.6 nm, and CdSe/ZnS quantum dots are used as a sensitizer with a diameter of 6 nm, where poly(methyl methacrylate) (PMMA) is used as the surrounding medium. The area of the cell is 50 nm × 50 nm, as shown in Figure 1d. The refractive index for MoTe_2_, CdSe/ZnS quantum dots, and SiO_2_ was taken from (refractiveindex.com) to determine the optical properties using COMSOL Multiphysics. Figure 1b shows the absorption of SiO_2_ when applied as a dielectric in photonic devices, with insignificant absorption between the visible and NIR spectral ranges because of its wide bandgap. These findings are in line with reports of thermally grown SiO_2_ substrates used widely today in optical interference and dielectric engineering [43,44].

Figure 1a presents the absorption spectrum of MoTe_2_, showing an absorption peak at 1170 nm, confirming its near-infrared (NIR) absorption capability. This observation is consistent with literature reports on few-layer and phase-engineered MoTe_2_ photodetectors exhibiting strong NIR absorption [45]. The absorption spectrum of CdSe/ZnS QDs (Figure 1c) shows discrete excitonic peaks arising from strong quantum confinement in the core–shell nanostructure. The absorption peak at approximately 650 nm is in good agreement with experimentally obtained spectra of QDs, validating the effective-medium Lorentz oscillator model employed in our optical simulations [46,47]. The optical equations of the SiO_2_/MoTe_2_/CdSe/ZnS heterostructure were simulated in the frequency domain by Maxwell equations. Figure 1e–g present the absorption, transmission, and reflection spectra of the complete heterostructure. The absorption spectrum (Figure 1e) shows a peak at 440 nm, which decreases with increasing wavelength. The transmission spectrum (Figure 1f) exhibits a complex behavior: transmission increases from 350 nm to 440 nm, then decreases around 600 nm, and subsequently increases at longer wavelengths. Figure 1g shows that reflectance increases with wavelength, exhibiting a peak at 440 nm and continuing to rise at longer wavelengths. Reflection increases in the SiO_2_/MoTe_2_/CdSe/ZnS QD heterostructure simulated in COMSOL primarily due to the sharp dielectric mismatches (mismatches in the refractive index) at the interfaces between the different layers. SiO_2_ has a relatively low refractive index (1.46) [48,49], while the refractive index of MoTe_2_ and CdSe/ZnS QDs is 4.789 (refractive index.info) and 2.4 [49]. The energy band diagram, obtained from COMSOL simulations, is shown in Figure 1h. The energy band alignment plot confirms the formation of a type-I heterostructure. The obtained bandgaps from left to right correspond to SiO_2_, MoTe_2_, and QDs; their values are 8.9 eV, 1.1 eV, and 2.1 eV. The MoTe_2_ layer possesses a lower conduction band (CB) and a higher valence band (VB) relative to the QDs. Consequently, the energy offsets at both the CB and VB act as driving forces for carrier localization, effectively trapping both electrons and holes within the narrower-bandgap region. Moreover, the DFT computations conducted with the help of Quantum Espresso also give additional insights into the electronic properties of materials. In the case of SiO_2_, the electron effective mass was found to be 0.47 m_0_, the electron mobility was estimated to be about 15 cm^2^ V^−1^ s^−1^, and the bandgap was 8.1 eV. Literature reports on thermally grown SiO_2_ show slightly reduced effective masses (0.42 m_0_) and reduced room-temperature electron mobilities, implying that the relatively higher mobility here could be due to approximation errors in the simulator [50,51].

For MoTe_2_ (2H), the calculated effective mass (*m*^⁎^ is 0.47 m_0_), mobility (180 cm^2^·V^−1^·s^−1^), and bandgap (0.88 eV) are in good agreement with previous DFT and experimental studies, which report effective masses around 0.7 m_0_, a bandgap of 1.07 eV, and carrier mobilities up to ~178 cm^2^·V^−1^·s^−1^ in optimized devices [51,52]. For the CdSe/ZnS quantum dot system, we obtained an effective mass *m^⁎^* of 0.13 m_0_, a mobility of 2 cm^2^·V^−1^·s^−1^, and a bandgap of ~1.8 eV. These values are consistent with literature reports for CdSe, where the electron effective mass is ~0.13 m_0_, and carrier mobility in QD systems is significantly reduced due to quantum confinement and surface scattering effects [53,54]. The errors in extracted electronic parameters as compared to the DFT literature values are mostly due to defects, dimensionality, quantum confinement, and non-band theory mechanisms of transport. Together with optical and band alignment studies, these data give a consistent physical picture of the SiO_2_/MoTe_2_/CdSe/ZnS heterostructure, which is why the structure should be considered in the context of high-responsivity broadband photodetector devices.

### 2.2. Electrical Properties

**Figure 2:**
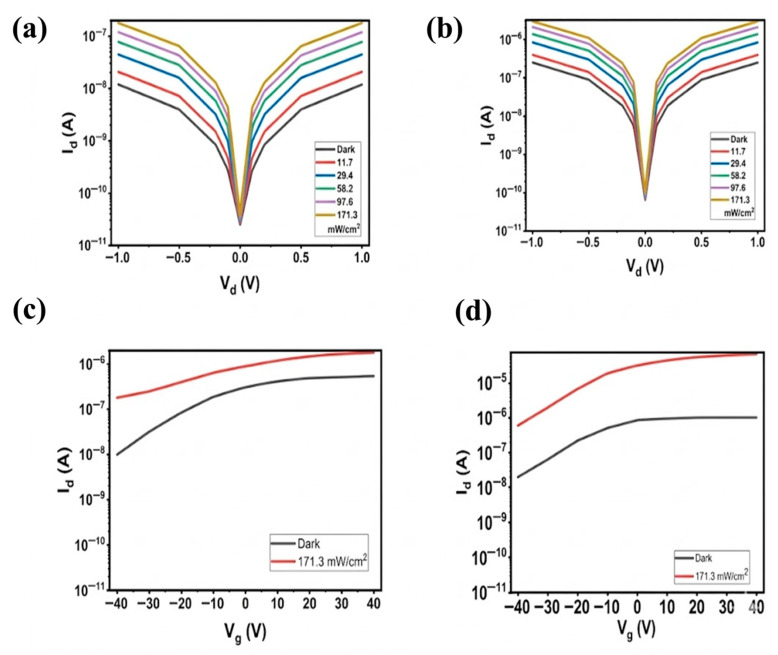
Simulated electrical characteristics of MoTe_2_-based photodetectors with and without CdSe/ZnS QDs when exposed to 520 nm illumination. (**a**) The output curve of the MoTe_2_-based photodetector without QDs shows its response to dark conditions and power density from 11.3 to 171.3 mW/cm^2^ at a 520 nm wavelength. (**b**) Output curves of the MoTe_2_-based photodetector with QDs under dark conditions and illumination power densities from 11.3 mW/cm^2^ to 171.3 mW/cm^2^ at a 520 nm wavelength. (**c**) Transfer curves of the MoTe_2_-based photodetector without QDs under dark and an illuminated power density of 171.3 mW/cm^2^ at 520 nm (V_d_ = 1 V). (**d**) Transfer curves of the MoTe_2_-based photodetector with QDs under dark and an illuminated power density of 171.3 mW/cm^2^ at 520 nm (V_d_ = 1 V).

Figure 2 shows the simulated electrical properties of MoTe_2_-based photodetectors under dark and illuminated conditions, obtained using Silvaco TCAD. These results demonstrate the impact of CdSe/ZnS QD integration on device performance. Figure 2a,b show the output characteristics (I_d_-V_d_ curves) of pristine MoTe_2_ and CdSe/ZnS QDs/MoTe_2_ heterostructure devices, respectively, measured at a constant gate voltage (V_g_ = 0 V) under varying optical power densities ranging from dark conditions to maximum illumination (171.3 mW/cm^2^). Figure 2a shows that the MoTe_2_ device has a monotonic dependence of drain current on drain voltage. The incident optical power modulates the drain current, demonstrating that photon absorption generates electron–hole pairs and enhances the drain current relative to the dark current. The curves are nearly linear, suggesting the existence of ohmic contact between MoTe_2_ and the electrodes of Au/Cr, which facilitates efficient carrier injection and results in photoconductive characteristics [55,56]. The net photocurrent with and without QDs is shown in Table 1.

Figure 2b represents a qualitatively similar trend in the QD-sensitized MoTe_2_ device with two distinct differences: a higher photocurrent when illuminated, as in Table 1. With increasing light, photogenerated carriers in the QD layer are transferred into the MoTe_2_ channel efficiently, leading to a much stronger drain current increase than in a pristine device. Such an action demonstrates the efficiency of the QD/MoTe_2_ heterostructure in encouraging the separation of charges and inhibiting recombination. Figure 2c,d show the transfer characteristics (I_d_-V_g_ curves) of pristine and QDs-integrated MoTe_2_ devices, respectively, measured at a constant drain voltage of V_d_ = 1 V. Figure 2c indicates that in the dark, the I_d_ varies with V_g_, exhibiting typical n-type semiconductor transfer characteristics. As a pure material, MoTe_2_ is an n-type semiconductor; i.e., it contains more electrons than holes as majority carriers. The drain current is larger in the whole range of gate voltages under illumination. Upon being illuminated, the photons form electron–hole pairs. In n-type MoTe_2_, photogenerated electrons serve as the majority carriers, contributing significantly to the photocurrent and resulting in increased drain current under illumination. Figure 2d, in the dark condition, the current through the drain is strongly dependent on the voltage of the gate, but the drain current is higher than that of the device in the absence of QDs, as shown in Figure 2. This is due to the strong light absorption of QDs and because effective charge separation at the interface contributes to an amplified photocurrent in the device. This particular behavior is observed in heterostructure photodetectors with 2D/quantum dot combinations, as many earlier works report [55]. These tendencies correspond to previously reported hybrid 0D/2D heterostructure photodetectors and demonstrate that QD sensitization significantly enhances the optoelectronic performance of MoTe_2_-based photodetectors by enabling efficient charge separation and transfer across a type-I band-aligned interface, with electrons moving to the n-type MoTe_2_. The separation and the low recombination rate of the photogenerated carriers result in an increased photocurrent during illumination. This can be observed in the large difference in the drain current when the device is in illuminated conditions as opposed to the MoTe_2_ device in the absence of QDs. The synergistic performance of MoTe_2_ and QDs proves the high potential of hybrid structures in high-performance photodetectors.

**Figure 3:**
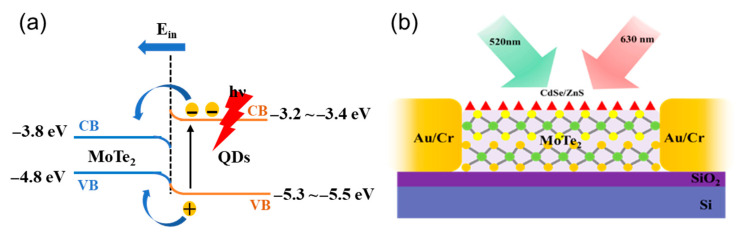
(**a**) Energy band diagram of the CdSe/ZnS/MoTe_2_-based photodetector [57], The blue lines in the figure represent the edge positions of MoS_2_, and the orange lines represent the edge positions of QDs. (**b**) Schematic of the complete device.

The energy band diagram of QDs/MoTe_2_ heterostructures is shown in Figure 3a. Previous work reports the conduction and valence bands of MoTe_2_ to be −3.80 and −4.80 eV (relative to the vacuum level), respectively [55]. In the case of QDs, the conduction and valence bands are reported to be −3.2~−3.4 eV and −5.3~−5.5 eV (relative to the vacuum level) [57]. Upon photoexcitation, photogenerated excitons are produced in the QD layer and separated by the built-in electric field (E_in_) at the interface. Due to band alignment, both carriers are injected into the MoTe_2_ layer. Electrons transfer from the CB of the QDs to MoTe_2_, while holes transfer from the deeper VB of the QDs to the higher VB of MoTe_2_. This efficient carrier injection modulates channel conductivity, enhancing the device’s optoelectronic performance. Figure 3b presents the schematic of the device, in which a monolayer of 0.6 nm MoTe_2_ and 6 nm QDs are used as a channel in the mixed-dimensional FET (field-effect transistor) with Au/Cr (100 nm/5 nm) being used for source/drain contacts on the back-gate Si/SiO_2_ (285 nm) substrate using Silvaco TCAD.

**Figure 4:**
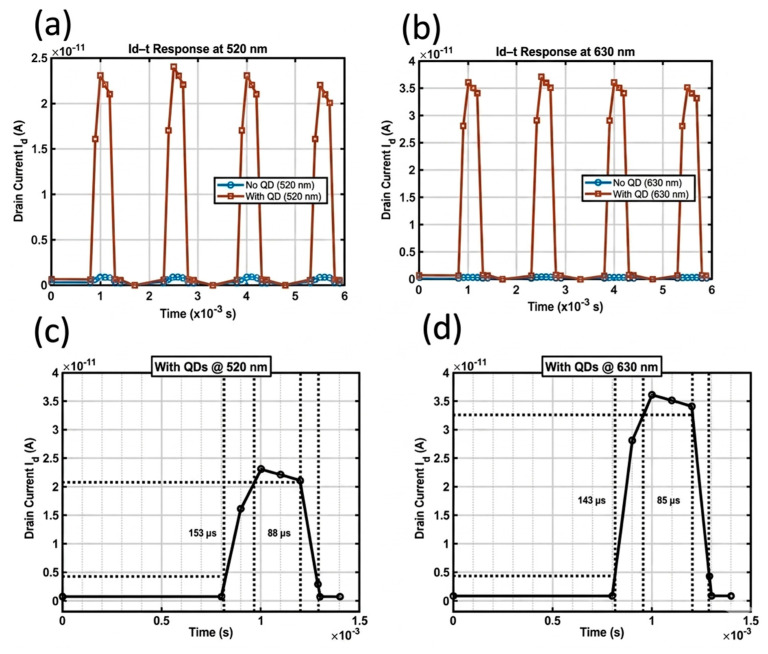
Simulated photoresponses of MoTe_2_ and CdSe/ZnS/MoTe_2_ heterostructure-based devices. (**a**) Time-dependent photoresponses of MoTe_2_ and CdSe/ZnS/MoTe_2_ under V_d_ = +1 V and V_g_ = 0 V, under 520 nm light illumination using an optical power density of 171.3 mW/cm^2^. (**b**) Time-dependent photoresponses of MoTe_2_ and CdSe/ZnS/MoTe_2_ at V_d_ = +1 V and V_g_ = 0 V, under 630 nm light illumination using the same power density. (**c**,**d**) Rise and decay times with QDs at 520 nm and 630 nm, the dashed line in the figure is used to determine the rise and decay time.

Figure 4 shows the Silvaco TCAD-modeled transient photoresponse of pristine MoTe_2_ and CdSe/ZnS QD-sensitized MoTe_2_ at 520 nm and 630 nm wavelengths at V_d_ = +1 V and V_g_ = 0 V with an optical power density of 171.3 mW/cm^2^. The photoresponse at 520 nm and 630 nm was reliably and reproducibly demonstrated with the device. The pristine MoTe_2_ exhibits a weak photoresponse at 520 nm and 630 nm compared with the QD-sensitized MoTe_2_, indicating weaker visible-light absorption and trap-dominated carrier dynamics in MoTe_2_. Moreover, the rise and decay times are determined from 10% to 90% and vice versa, as shown in Figure 4c,d. The rise and decay times at 520 nm were 168 and 209 μs, respectively, and with the addition of QDs, the rise and decay times were reduced to 153 and 88 μs, which means a general decrease in the response time and more balanced carrier dynamics. The rise and decay times at 630 nm were 154 and 177 μs on bare MoTe_2_, and the rise and decay times decreased to 143 and 85 μs with the addition of QDs, indicating that response speed is also enhanced. The ZnS shell of the QDs plays a critical cleaning role on the MoTe_2_ surface by passivating surface states such as oxygen vacancies and defects that normally act as carrier traps [55]. These traps typically produce a slow tail in the transient response because carriers become trapped and are released gradually. At 520 nm illumination, the light has a short penetration depth and generates carriers near the surface where these traps are concentrated, so passivation by the QDs effectively cleans up the surface and leads to a faster response. Furthermore, the type-I heterojunction between QDs and MoTe_2_ also allows an improvement in the response time. In comparison, 630 nm light penetrates deeper into the MoTe_2_ flake, generating carriers mainly in the bulk region far from the surface-passivated interface. The transient behavior is governed primarily by bulk carrier recombination and relaxation processes inside MoTe_2_, where the surface passivation effect of the QDs becomes much less important. Therefore, the improvement in response time is significant at 520 nm as compared to 630 nm [58]. As illustrated in Figure 4a,b, the MoTe_2_ device sensitized with CdSe/ZnS QDs exhibits a significant enhancement in photocurrent compared to the pristine device. Under 520 nm illumination, the photocurrent increases from 6 × 10^−13^ to 2.24 × 10^−11^ A, while at 630 nm, it rises from 2.8 × 10^−13^ to 3.53 × 10^−11^ A. This improvement of nearly two orders of magnitude is attributed to the efficient separation of photogenerated excitons within the QDs and the subsequent carrier injection into the MoTe_2_ channel, which increases the channel conductivity. The simulated heterostructure replicates the important performance patterns of state-of-the-art broadband QD-sensitized phototransistors, compared with previously reported devices only using MoTe_2_, and proves that QD integration can effectively overcome the inherent absorption and recombination limitations of MoTe_2_ by the physical model used in the TCAD simulation framework.

To thoroughly assess the performance of the photodetector, using simulated results obtained from Silvaco TCAD, we calculated essential parameters such as responsivity (*R*), specific detectivity (*D*), and external quantum efficiency (*EQE*) via the subsequent formulas:

(1) $$R=\frac{I_{ph}}{P\times A}$$

(2) $$D=R\times \sqrt{\frac{A}{2qI_{dark}}}$$

(3) $$EQE=\;\frac{hcR}{q\lambda }\times 100\%$$

In these calculations, *I_ph_* is used to denote the photocurrent produced under power densities, and *I_dark_* represents the dark current produced in the absence of light. The incident optical power density is referred to as P_in_. The area of the illuminated object of the device used in this case is 1 × 10^−7^ cm^2^ and denoted by A. *q* is the elementary charge of an electron, *h* is the Planck constant, *c* is the speed of light, and *λ* is the wavelength of light radiation [56,59,60]. These parameters are essential for determining the key performance metrics of the photodetector, including *R*, *D*, and *EQE*. The power density at 520 nm and 630 nm = 171.3 mW/cm^2^, with V_d_ = 1 V, V_g_ = 0 V. The CdSe/ZnS/MoTe_2_ heterostructure exhibited excellent operation in the wavelengths between 520 nm and 630 nm. At 520 and 630 nm, a responsivity of 1.3 × 10^−3^ A/W and 2 × 10^−3^ A/W, a detectivity of 9.4 × 10^8^ Jones and 1.34 × 10^9^ Jones, and an EQE of 0.31% and 0.394% were achieved with the CdSe/ZnS/MoTe_2_ heterostructure. MoTe_2_ had a responsivity of 3.5 × 10^−4^ A/W and 2.8 × 10^−4^ A/W, a specific detectivity of 1.13 × 10^8^ Jones and 1.11 × 10^8^ Jones, and an *EQE* of 0.0835% and 0.055% at 520 nm and 630 nm, respectively. These significant improvements in the *EQE*, detectivity, and photoresponsivity reflect the significance of integrating QDs in MoTe_2_-based photodetectors. These findings demonstrate QDs/MoTe_2_ photodetectors’ potential in photonics applications, reflecting the advantages of excellent improvements in light absorption, charge carrier dynamics, and the overall photodetector performance. Future studies will have the objective of refining material interfaces even more and considering other methods of photodetection other than the ones presently in use. Also, we compared the efficiency of our novel MoTe_2_ and QDs/MoTe_2_ photodetectors with conventional photodetection techniques, as can be seen in Table 2. The findings are that our devices’ performance will be either better or comparable to existing photodetectors. Such outstanding photoresponse properties can be explained by the proper design of the device structure and the synergetic effects between the materials employed.

## 3. Conclusions

In short, a fully integrated model of CdSe/ZnS quantum dot-sensitized MoTe_2_ photodetectors, combining electromagnetic modeling, density functional theory, and device-scale Silvaco TCAD, is presented in this work. It is shown through optical studies that integrating CdSe/ZnS QDs into the heterostructure of SiO_2_/MoTe_2_ is a very convenient method of increasing light absorption in the visible range. Calculation of band structure supports the presence of type-I band alignment at the CdSe/ZnS/MoTe_2_ interface, which allows a high level of interfacial charge separation and no recombination of carriers. Transient photoresponse and electrical simulations demonstrate that the incorporation of QDs offers a significant performance enhancement for photodetectors via current photogating and interfacial charge transfer processes. At 520 and 630 nm, the CdSe/ZnS/MoTe_2_ heterostructure demonstrates a significantly improved photocurrent, responsivity, detectivity, and external quantum efficiency compared to pristine MoTe_2_ devices. The responsivity (1.3 × 10^−3^ A/W), detectivity (9.4 × 10^8^ Jones), and *EQE* (0.31%) show that QD sensitization would enable the natural absorption and recombination limits of MoTe_2_ to be overcome. Several experimental challenges must be addressed to transform these theoretical findings into practical devices. First, achieving a pristine, defect-free interface between the 0D QDs and the 2D MoTe_2_ flake is critical, as interfacial contaminants during the solution processing of QDs can introduce trap states that hinder charge transfer. Second, precise control over the QD deposition density and the length of the organic capping ligands is essential; while shorter ligands facilitate faster charge injection, they may also affect colloidal stability and film uniformity. Finally, the environmental stability of MoTe_2_ remains a concern for long-term operation. Future research should focus on optimizing interface passivation techniques and exploring encapsulation strategies to enhance both the sensitivity and operational lifespan of these hybrid low-dimensional optoelectronic systems.

## 4. Experimental Section

A multiscale simulation, which is a combination of first-principles, optical, and device-level simulations, was used to study the CdSe/ZnS QDs/MoTe_2_-based photodetector. The MoTe_2_, CdSe/ZnS QDs, and SiO_2_ band structure, effective masses, and carrier mobilities were determined using density functional theory (DFT) calculations with the help of Quantum ESPRESSO. The heterostructure of interest was a SiO_2_ (285 nm)/MoTe_2_ (0.6 nm)/CdSe/ZnS QDs (~6 nm) stack, simulated in COMSOL Multiphysics (Wave Optics Module) to study the optical response of the heterostructure. The geometry was assembled in the form of a stacked structure, where SiO_2_ was the dielectric, MoTe_2_ was the active material, and CdSe/ZnS QDs were the excitonic absorber. It was assumed that the QD layer was an effective medium, and a Lorentz oscillator was used to recreate the strong excitonic resonance at 650 nm. The database of refractive index (refractiveindex.com) imported wavelength-dependent complex refractive indices *n + ik* of SiO_2_, MoTe_2_, CdSe/ZnS. The frequency-domain solution of the model was done via finite element discretization, and convergence was achieved through mesh refinement (6 nm) of the monolayer of MoTe_2_. Boundary conditions had a plane-wave input port, an output transmission port, and perfectly matched layers (PMLs) to ensure artificial reflections were suppressed. Integrated Poynting flux at the ports was measured as reflections (*R*) and transmission (*T*) spectra, and absorption (*A*) was calculated as

*A*(*λ*) = 1 − *R*(*λ*) − *T*(*λ*) (4)

where *A*(*λ*), *R*(*λ*), and *T*(*λ*) denote the wavelength-dependent absorption, reflection, and transmission, respectively. Local absorption distributions were extracted via

(5) Q(r,λ)=w2ε0Imεγ,λE(r,λ)2

where *w* is the angular frequency of incident light, *ε*_0_ is the vacuum permittivity, Im[*ε*(*r*, *λ*)] is the imaginary component of the relative permittivity, and *E*(*r*, *λ*) is the amplitude of the local electric field. The electrical properties were analyzed in the Silvaco TCAD by specifying the structure of the device and simulating dark and light I-V features. The results of the optical generation profiles of COMSOL were added to the TCAD model to evaluate the photocurrent, responsivity, and detectivity, and to give a detailed picture of the work of the photodetector. There were clear Fabry–Pérot interference fringes in the simulated spectra, both in the background reflection and transmission in the visible range, because of the SiO_2_ spacer between the layers. The reflectance spectrum (Figure 1g) peaks at 440 nm and shows continuous variation with wavelength, further confirming interference modulation caused by refractive index mismatch and cavity effects. The nonlinear transmission (Figure 1f) increases and dips near 600 nm, then increases again, directly indicating wavelength-selective constructive and destructive interference in the (SiO_2_) layer. A sharp excitonic absorption peak was observed at approximately 650 nm, in line with the size-dependent bandgap of 6 nm CdSe/ZnS QDs, while the MoTe_2_ layer exhibits near-infrared absorption at around 1170 nm, in line with its direct bandgap. The absorption map indicated by spatial absorption measurements revealed that visible-light absorption was mainly concentrated in the QD layer, and MoTe_2_ was concentrated in the longer-wavelength range. Notably, the combination of QDs and MoTe_2_ absorption edges also increased the overall device absorption, facilitating the production of excitons and inter-layer interaction. Equally, we find that electrons excited in CdSe conduction band states can be transferred to MoTe_2_ and holes left in the QD valence band; thus, spatial carrier separation is achieved, which is advantageous for photodetection.

## Figures and Tables

**Table 1:** Net photocurrent response as a function of incident power density for devices measured without and with CdSe/ZnS quantum dot sensitization.

Power Density (mW/cm^2^)	Net Photocurrent w/o CdSe/ZnS (A)	Net Photocurrent w/CdSe/ZnS (A)
11.7	9 × 10^−9^	1.50 × 10^−7^
29.4	3.30×10^−8^	6 × 10^−7^
58.2	6.60 × 10^−8^	1.15 × 10^−6^
97.6	1.08 × 10^−7^	1.85 × 10^−6^
171.3	1.68× 10^−7^	2.75 × 10^−6^

**Table 2:** Performance comparison of photodetectors: our findings vs. reported 2D material-based heterostructures.

Device	Wavelength (nm)	Responsivity (A/W)	Detectivity (Jones)	EQE (%)	Ref.
MoTe_2_	520	3.5 × 10^−4^	1.13 × 10^8^	0.0835	This Work
MoTe_2_	630	2.8 × 10^−4^	1.11 × 10^8^	0.055	This Work
CdSe/ZnS QDs/MoTe_2_	520	1.3 × 10^−3^	9.4 × 10^8^	0.31	This Work
CdSe/ZnS QDs/MoTe_2_	630	2 × 10^−3^	1.34 × 10^9^	0.394	This Work
Graphene/ZnO NW heterostructure	325	3.5 × 10^− 6^	-	-	[61]
WSe_2_/ZnO heterostructure	300	4.5 × 10^−2^	~10^9^	-	[62]
ZnS–MoS_2_	554	1.78 × 10^−5^	-	0.4	[63]
MoTe_2_/MoS_2_	473	4.6 × 10^−3^	-	-	[64]
ReS_2_/ReSe_2_	550	2.1 × 10^−2^	-	4.76	[65]
MoTe_2_/MoS_2_	470	3.22 × 10^−2^	-	-	[66]
MoSe_2_	532	1.7 × 10^−4^	1.158 × 10^9^	0.025	[60]
Graphene/Si	390	1.8 × 10^−1^	-	-	[67]
MoS_2_–MoO_3_	405	1.3 × 10^−4^	-	0.041	[68]
MoTe_2_/CdS/ReS_2_	450	4.92 × 10^2^	3.12 × 10^15^	1.04 × 10^5^	[69]
ZnO/P(EDOS-T)-1 cycle	365	1.54 × 10^−1^	4.2 × 10^9^	-	[70]
GaN nanowire/Nb-doped MoS_2_	500	3 × 10^−1^	-	-	[71]
PbSeQDs/MoTe_2_/MoSe_2_	635	1.183 × 10^4^	-	-	[72]
ZnOQDs/POM	-	1.835 × 10^−1^	-	-	[73]
WSe_2_/CsPbBrIQs GeSe/MoTe_2_	638 638	9.66 × 10^1^ 1.98	1.25 × 10^11^ 1.15 × 10^11^	- 387	[74,75]
MoS_2_/graphene/WSe_2_	520	7.8 × 10^3^	8.7 × 10^13^	-	[76]
WS_2_NP/MoS_2_	580	2.8 × 10^−1^	6.44 × 10^12^	61	[77]
MoS_2_/ZnCdSeQDs	<700	3.7 × 10^4^	1 × 10^12^	-	[78]
ZnOQDs/WSe_2_	visible	1.0 × 10^4^	1.98 × 10^11^	-	[79]

## Data Availability

The data underlying this study’s findings can be obtained from the corresponding author upon reasonable request.
